# Dietary Intake of Polyunsaturated Fatty Acids Is Associated with Blood Glucose and Diabetes in Community-Dwelling Older Adults

**DOI:** 10.3390/nu16234087

**Published:** 2024-11-27

**Authors:** Hélio José Coelho-Júnior, Alejandro Álvarez-Bustos, Anna Picca, Riccardo Calvani, Leocadio Rodriguez-Mañas, Francesco Landi, Emanuele Marzetti

**Affiliations:** 1 Fondazione Policlinico Universitario Agostino Gemelli IRCCS, L.go A. Gemelli 8, 00168 Rome, Italy; picca@lum.it (A.P.); riccardo.calvani@unicatt.it (R.C.); francesco.landi@unicatt.it (F.L.); 2 Department of Geriatrics, Orthopedics and Rheumatology, Università Cattolica del Sacro Cuore, L.go F. Vito 1, 00168 Rome, Italy; 3 Department of Geriatrics, Hospital Universitario de Getafe, Ctra de Toledo, 28905 Getafe, Spain; leocadio.rodriguez@salud.madrid.org; 4 Biomedical Research Center Network for Frailty and Healthy Ageing (CIBERFES), Institute of Health Carlos III, Av. Monforte de Lemos 3–5, 28029 Madrid, Spain; a.alvarezbu@gmail.com; 5 Instituto de Investigación Hospital Universitario La Paz (IdiPaz), C. de Pedro Rico 6, 28029 Madrid, Spain; 6 Department of Medicine and Surgery, LUM University, Strada Statale 100 km 18, 70010 Casamassima, Italy

**Keywords:** malnutrition, metabolic syndrome, obesity, omega-3, omega-6

## Abstract

Objectives: The present study was conducted to examine the cross-sectional associations between the dietary intake of polyunsaturated fatty acids (PUFAs) and cardiometabolic risk factors in a large sample of Italian community-dwelling older adults. Methods: This is a cross-sectional study. Longevity Check-up 8+ (Lookup 8+) is an ongoing project that started in June 2015. The project is conducted in unconventional settings (e.g., exhibitions, malls, and health promotion campaigns) across Italy with the aim of fostering the adoption of healthy lifestyles in the general population. For the present study, participants were eligible if they were 65+ years and provided written informed consent. Systolic blood pressure (SBP) and diastolic blood pressure (DBP), and blood glucose and cholesterol levels were assessed. A 12-item food frequency questionary was used to estimate the dietary intake of PUFAs, which included omega-3 (α-linolenic acid [ALA], eicosapentaenoic acid [EPA], and docosahexaenoic acid [DHA]) and omega-6 fatty acids. Results: Data of 4461 older adults (♀56%, mean age: 72.9 years, mean body mass index [BMI]: 26.1 kg/m^2^, blood glucose: 109 mg/dL, total blood cholesterol: 198.5 mg/dL, ALA: 8.8%, EPA: 16.0%, and DHA: 26.1%) were cross-sectionally analyzed. Multilinear regression results indicated that a high consumption of EPA, DHA, and DHA+EPA was negatively and significantly associated with glucose levels. Furthermore, binary regression analysis indicated that the dietary intake of ALA and omega-6 PUFAs was inversely and significantly associated with the prevalence of diabetes. In contrast, BMI values were positively associated with ALA and omega-6 PUFAs, and negatively with the consumption of EPA and DHA+EPA. Conclusions: Findings of the present study indicate that the dietary intake of PUFAs was cross-sectionally, inversely, and significantly associated with blood glucose levels and the prevalence of diabetes in a large sample of Italian community-dwelling older adults.

## 1. Introduction

Cardiovascular disease (CVD) is the leading global cause of mortality, claiming nearly 20 million lives each year [1]. Older adults are disproportionately affected, with approximately 70% of individuals aged 65 and older presenting with cardiometabolic risk factors [2,3]. This elevated burden among older populations is particularly concerning, as it is linked to adverse outcomes such as increased rates of hospitalization, higher healthcare expenditures, and elevated mortality risks [2,3]. The onset of CVD in late life often results from disruptions in cardiometabolic regulation that occur with aging [4]. These dysregulations typically manifest as elevated blood pressure, impaired glucose metabolism, and lipid imbalances.

Diabetes is a major risk factor for the development of CVD [1]. The current epidemiological data indicate that more than 500 million people are living with diabetes worldwide [5], among whom 4 million are estimated to be Italians, representing 10% of the adult population [6]. Alongside its association with CVD, the progression of diabetes is commonly associated with an increased risk of microvascular complications (i.e., retinopathy, neuropathy, and nephropathy), promoting severe damage in vital organs (e.g., kidney) and contributing to the development of other chronic conditions (e.g., chronic kidney disease), and, finally, early death [7]. In Italy, diabetes-related complications (e.g., hospital admissions, drugs, outpatient care) produce high costs to the public treasury, around €9 billion every year [8].

Adherence to healthy lifestyle habits is an established strategy to improve cardiometabolic health [9]. Polyunsaturated fatty acids (PUFAs) are essential fatty acids almost exclusively obtained from diet sources [10,11]. Omega-3 is one of the main classes of PUFAs and refers to three main types of fatty acids: α-linolenic acid (ALA), eicosapentaenoic acid (EPA), and docosahexaenoic acid (DHA) [10,11]. These molecules are essential in cell signaling and structure, with considerable attention given to their anti-inflammatory properties due to the potential impacts on cardiometabolic risk factors, such as insulin resistance [12]. Despite the plausibility behind these assumptions, more studies are needed before definitive conclusions can be reached on the cardiometabolic benefits of omega-3 [12,13].

The need for more data on the subject is emphasized by a recent systematic review and meta-analysis that found conflicting results on the association between the dietary intake of PUFAs and diabetes [14]. Moreover, authors observed that the positive effects of PUFA consumption was restricted to Asian populations [14]. However, no studies examining Mediterranean populations were included in the pooled analysis. This scenario warrants attention given that people living in Mediterranean countries, such as Italians, for example, often show a moderate-to-high adherence to diet patterns rich in omega-3 PUFAs [15,16]. Furthermore, just a few investigations have examined samples composed exclusively of older adults.

To expand the knowledge on this subject, the present study was conducted to examine the cross-sectional associations between the dietary intake of PUFAs and cardiometabolic risk factors in a large sample of Italian community-dwelling older adults.

## 2. Materials and Methods

This investigation is based on a cross-sectional design. Data were drawn from the Longevity Check-up 8+ (Lookup 8+) project database, a continuing initiative managed by the Department of Geriatrics at Fondazione Policlinico “Agostino Gemelli” IRCCS and Università Cattolica del Sacro Cuore, located in Rome, Italy. Details regarding participant recruitment, procedures, and outcomes have been described in previous publications [17,18,19]. The study adhered to the ethical principles outlined in the Declaration of Helsinki and received approval from the Ethics Committee of Università Cattolica del Sacro Cuore (protocol number A.1220/CE/2011). This report was prepared in compliance with the STROBE guidelines for observational studies [20].

### 2.1. Participants

From 1 June 2015 to 31 December 2023, the study included 17,576 adults aged 18 years and older living in the community. Participants were excluded if they were unable or unwilling to provide written informed consent, reported being pregnant, or were unable to complete physical function assessments as specified in the study protocol. For this specific analysis, only individuals aged 65 and older with a body mass index (BMI) of at least 18.5 kg/m^2^ and complete data were included, yielding a final sample of 4461 participants. A total of 13,115 individuals were excluded, of whom 8232 were under the age of 65.

### 2.2. Data Collection

Data collection involved obtaining information on participants’ lifestyle habits, alongside physical measurements of height and weight. BMI was calculated by dividing weight in kilograms by the square of height in meters (kg/m^2^). Health conditions were identified based on participants’ self-reports. Blood pressure measurements, including systolic and diastolic values, were taken with an oscillometric monitor (Omron M6, Omron, Kyoto, Japan). Glucose and total cholesterol levels were assessed using capillary blood samples analyzed by a portable device (MultiCare-In, Biomedical Systems International Srl, Florence, Italy), which adheres to the UNI EN ISO 15197:2015 guidelines for accuracy, safety, and usability in glucose monitoring (https://store.uni.com/en-iso-15197-2015, accessed on 15 October 2024).

Information on fasting status was gathered by asking participants whether they had refrained from eating for at least eight hours before the assessments. Smoking behavior was categorized based on lifetime smoking history, with individuals who had smoked at least 100 cigarettes and were currently smoking classified as smokers, while others were labeled as non-smokers. Physical activity levels were evaluated by examining engagement in exercise routines, which allowed participants to be categorized as either active or inactive.

Dietary patterns were evaluated using a food frequency questionnaire (FFQ), which documented the weekly intake for 12 food categories. Standard portion sizes were based on nutritional benchmarks provided by Italian dietary references [21]. To calculate daily averages for nutrients such as calories, proteins, omega-6 fatty acids, ALA, EPA, and DHA, consumption frequencies were multiplied by the nutrient content of standard servings and averaged over seven days. Omega-3 fatty acid intake was quantified by summing the values of ALA, EPA, and DHA.

### 2.3. Statistical Analysis

The Shapiro–Wilk test was used to assess the normality of variable distribution. Continuous variables are presented as means ± standard deviations (SD), while categorical variables are reported as percentages. Unadjusted and adjusted linear regressions were applied to examine associations between PUFA intake and blood pressure and glucose and cholesterol levels. Binary logistic regressions (unadjusted and adjusted) were run to analyze the link between PUFA intake and the prevalence of hypertension, diabetes, and obesity. The final model adjusted for age, sex, BMI, energy intake, fasting state (for glucose), physical activity, and smoking status. Results are reported as unstandardized coefficients (β) with 95% confidence intervals (CI). Data were rounded to two decimal places, with significance set at *p* < 0.05. All analyses were conducted using SPSS software (version 23.0, SPSS Inc., Chicago, IL, USA).

## 3. Results

### 3.1. Participants

The data of 4461 older adults (women = 2597, 56%) were analyzed. The main characteristics of study participants are shown in Table 1. Participants had a mean (SD) age of 72.9 ± 6.0 years and an average BMI of 26.1 ± 4.0 kg/m^2^, suggesting that most of them were within normal weight or were overweight. The prevalence of hypertension, diabetes, and obesity was 21.0%, 15.5%, and 15.5%, respectively. When compared to data of the Italian population, our sample had a lower prevalence of diabetes [6], obesity [22], and hypertension [23]. Furthermore, the BMI and calorie intake values were virtually similar to the observations in the overall Italian population 18+ (25.6 kg/m^2^ and 2490 kcal) [24,25].

### 3.2. Polyunsaturated Fatty Acids and Cardiometabolic Risk Factors

The results of linear and multiple regressions are shown in Table 2. In the unadjusted analysis, the dietary intake of ALA and omega-6 PUFAs was significantly associated with systolic blood pressure, BMI, and glucose levels. ALA consumption was also associated with cholesterol levels. Moreover, BMI and glucose levels were significantly associated with the dietary intake of DHA, EPA (only glucose), and EPA+DHA.

After adjusting the analysis for covariates, the dietary intake of ALA and omega-6 PUFAs were significantly associated with BMI values. In contrast, EPA, DHA (only for glucose), and DHA+EPA consumption were negatively and significantly associated with BMI and glucose levels.

### 3.3. Polyunsaturated Fatty Acids and the Prevalence of Cardiometabolic Diseases

The results of the unadjusted and adjusted binary regressions are shown in Table 3. In the unadjusted analysis, the prevalence of hypertension, diabetes, and obesity were significantly associated with the dietary intake of ALA and omega-6 PUFAs. After adjusting the analysis for covariates, ALA and omega-6 PUFA consumption was negatively and significantly associated with the prevalence of diabetes. No other significant results were found.

## 4. Discussion

The main findings of the present study indicate that the dietary intake of PUFAs is cross-sectionally associated with diabetes. Specifically, a high consumption of EPA, DHA, and DHA+EPA was negatively and significantly cross-sectionally associated with glucose levels. Furthermore, binary regression analysis indicates that the dietary intake of ALA and omega-6 PUFAs is cross-sectionally, inversely, and significantly associated with the prevalence of diabetes. In contrast, BMI values were positively associated with ALA and omega-6 PUFAs, and negatively with the consumption of EPA and DHA+EPA.

Investigations have found mixed results regarding the associations between PUFAs, glucose levels, and the prevalence of diabetes. Takahashi et al. [26] found significant and positive associations between PUFAs and diabetes severity. In contrast, Marushka et al. [27] and Brostow et al. [28] observed that a high dietary intake of omega-3 was inversely associated with diabetes in middle-aged adults. To complicate things further, van Woudenbergh et al. [29] reported no significant associations between diabetes, DHA, and EPA in a Dutchpopulation-based study. These conflicting results were confirmed by a recent systematic review and meta-analysis that examined more than 50,000 individuals and found negative associations between the dietary intake of omega-6 and diabetes, whereas positive associations were observed for DHA consumption [14].

The different results observed among the studies are likely due to sample characteristics, study type (e.g., case–control, cross-sectional longitudinal), dietary intake assessment methods, portion sizes, geographical location, habitual and cultural consumption of PUFAs sources (e.g., fish), and covariates used for statistical adjustment, to quote a few possible variables.

The current cross-sectional study adds to the existing knowledge by providing information about a large sample composed exclusively of older adults. These data are particularly valuable, given that most studies examined samples combining young and middle-aged adults [14]. Analyzing mixed-age groups is complex and might cause erroneous interpretations. In fact, aging may be associated with increasing insulin resistance and a consequent decrease in glucose uptake, promoting the development of diabetes [30]. Moreover, older adults are more likely to display changes in appetite and physical activity levels [31,32]. As such, data referring to younger adults tend to be more homogenous, given the relatively mild variations in glucose control and behaviors, whereas more severe dysregulations are expected to be found in older adults.

Furthermore, our cross-sectional findings do not support the possibility raised by some authors that PUFA consumption is not inversely associated with diabetes in European populations [14,33], since Western dietary patterns are characterized by a high intake of sugar, red meat, and fried foods [14]. Notably, the present cross-sectional study examined Italian older adults, who typically show a moderate-to-high adherence to the Mediterranean diet [16]. This dietary pattern is expected to provide a high amount of omega-3 and involves a preferential intake of plant foods, and a moderate consumption of eggs, seafood and fish, and dairy products, in addition to a rare intake of red meat [15]. Moreover, olive oil is used as the most abundant source of fat and red wine is consumed with moderation [15].

The variation in the results might also be explained by the PUFA assessment method, which frequently includes FFQs, dietary recalls, and biomarkers. FFQs, for example, might vary in the number of items, the time frame of examination, and the inclusion, or not, of portion sizes [34]. In general, it is believed that a short FFQ may miss dietary intake variations, whereas an overly detailed instrument can be time-intensive, potentially affecting the data quality [34]. On the other hand, dietary recalls are open-ended questionaries in which participants detailed their habitual dietary intake for a determined period [34]. The major concern regarding this method is that individual days may not be representative of a person’s usual diet [34]. In addition, although biomarkers (i.e., plasma and erythrocytes) have been frequently used as proxies of the dietary intake of PUFAs and omega-3 [14], empiric evidence has only found low-to-moderate correlations among them [35,36].

The numerous possible mechanisms underlying the anti-diabetic effects of PUFAs have been extensively reviewed [37,38]. For instance, omega-3 might contribute to glucose homeostasis by inhibiting the activation of the toll-like receptor 4 (TLR4) pathway, thereby reducing the synthesis and activity of pro-inflammatory cytokines, ameliorating the insulin sensitivity and glucose uptake [39]. Improvements in fasting glucose levels and insulin resistance in response to omega-3 have also been associated with an increased expression of peroxisome proliferator-activated receptors (PPARs), mainly subunits α and γ, and of downstream proteins of the insulin signaling pathway, including the insulin receptor, the phosphatidylinositol-3-kinase (PI3K), and, finally, glucose transporter 4 (GLUT-4) [40].

We observed significant cross-sectional associations between omega-3 fatty acids and BMI values. These results could be attributed to the varying sources of these fatty acids, since ALA and omega-6 are found in plant oils, whereas EPA and DHA are found in fish and seafood [41]. Hence, it is possible that these associations might reflect general dietary patterns, instead of direct relationships. Moreover, the fact that the mean BMI values of our examined population were classified as normal weight/overweight and that no significant associations were observed with obesity suggests that the consumption of omega-3 PUFAs might be inversely associated with malnutrition. However, we currently lack the data to test this hypothesis, and further studies are needed.

The present cross-sectional study is not free of limitations. First, as the sample comprises relatively young, community-dwelling Caucasian older adults, caution is needed when generalizing findings to other populations or conditions. Second, the FFQ used in this study captured only the weekly intake of 12 food groups, potentially overlooking certain foods or leading to under-reported dietary habits. Third, important clinical markers related to glucose regulation, such as HbA1c levels, were not included. Fourth, data on specific blood glucose-lowering medications were not collected in the Lookup 8+ study. Fifth, some participants with diabetes might have been following dietary plans to manage blood sugar levels, potentially influencing results. Lastly, the cross-sectional design limits the ability to observe changes over time or establish cause–effect relationships among the studied variables.

## 5. Conclusions

The findings of the present cross-sectional study indicate that the dietary intake of PUFAs is inversely and significantly associated with glucose levels and the prevalence of diabetes in a large sample of Italian community-dwelling older adults. These findings add to the current knowledge by indicating that the dietary intake of PUFAs might contribute to the management of diabetes. However, longitudinal studies are required to confirm and expand our data.

## Figures and Tables

**Table 1:** Main characteristics of study participants (*n* = 4461).

Variable	Mean (SD) or %
Age, years	72.9 ± 6.0
Women	56.0
Weight, kg	70.6 ± 13.0
Height, m	1.63 ± 0.1
BMI, kg/m^2^	26.1 ± 4.0
Systolic blood pressure, mmHg	129.9 ± 16.1
Diastolic blood pressure, mmHg	75.9 ± 9.6
Blood glucose, mg/dL	109.0 ± 16.1
Total blood cholesterol, mg/dL	198.5 ± 35.0
Hypertension	21.0
Diabetes	15.5
Obesity	15.5
Calorie intake, kcal	2524.6 ± 760.0
Carbohydrates, g	218.4 ± 71.1
Lipids, g	153.1 ± 67.2
Proteins, g	177.6 ± 58.0
SFA	31.7
MUFAs	26.7
Omega-6	29.8
ALA	8.8
EPA	16.0
DHA	26.1
Smoking	14.0
Physically active	51.0

ALA = α-linolenic acid; BMI = body mass index; DHA = docosahexaenoic acid; EPA = eicosapentaenoic acid; MUFAs = monounsaturated fatty acids; SFAs = saturated fatty acids.

**Table 2:** Unadjusted and adjusted associations between PUFAs and cardiometabolic risk factors.

Variables	Unadjusted β	95% CI	*p*-Value	Adjusted β	95% CI	*p*-Value
*Systolic blood pressure*						
Omega-6	**0.04**	**0.01, 0.07**	**0.011**	0.01	−0,02, 0.05	0.338
ALA	0.24	**0.09, 0.38**	**0.001**	0.07	−0.08, 0.23	0.335
EPA	0.03	−0.02, 0.08	0.255	0.03	−0.02, 0.09	0.201
DHA	0.01	−0.01, 0.04	0.372	0.01	−0.01, 0.04	0.219
EPA+DHA	0.01	−0.01, 0.02	0.326	0.01	−0.07, 0.03	0.211
*Diastolic blood pressure*						
Omega-6	0.01	−0.01, 0.02	0.686	−0.01	−0.02, 0.02	0.955
ALA	0.02	−0.01, 0.11	0.534	−0.01	−0.10, 0.08	0.856
EPA	0.01	−0.02, 0.04	0.600	0.01	−0.02, 0.04	0.418
DHA	0.01	−0.01, 0.02	0.499	0.01	−0.01, 0.02	0.295
EPA+DHA	0.01	−0.01, 0.01	0.532	0.01	−0.01, 0.01	0.333
*Body mass index*						
Omega-6	**0.02**	**0.01, 0.03**	**0.001**	**0.02**	**0.01, 0.03**	**0.001**
ALA	**0.10**	**0.06, 0.13**	**0.001**	**0.07**	**0.03, 0.11**	**0.001**
EPA	**−0.01**	−0.02, 0.01	0.065	−0.01	−0.02, 0.01	0.111
DHA	**−0.01**	**−0.01, −0.03**	**0.007**	**−0.01**	**−0.01, −0.01**	**0.027**
EPA+DHA	**−0.01**	**−0.01, −0.01**	**0.017**	**−0.01**	**−0.01, 0.01**	**0.046**
*Blood glucose*						
Omega-6	**0.07**	**0.02, 0.12**	**0.005**	0.06	−0.01, 0.12	0.057
ALA	**0.32**	**0.09, 0.55**	**0.005**	0.09	−0.161, 0.348	0.472
EPA	**−0.11**	**−0.20, −0.23**	**0.014**	**−0.09**	**−0.185, −0.002**	**0.045**
DHA	**−0.07**	**−0.11, −0.21**	**0.005**	**−0.05**	**−0.104, −0.005**	**0.030**
EPA+DHA	**−0.04**	**−0.07, −0.01**	**0.007**	**−0.03**	**−0.067, −0.003**	**0.034**
*Total blood cholesterol*						
Omega-6	−0.01	−0.07, 0.06	0.889	−0.02	−0.105, 0.005	0.622
ALA	**−0.54**	**−0.84, −0.23**	**0.001**	**−0.25**	−0.594, 0.085	0.141
EPA	0.05	−0.06, 0.18	0.362	−0.01	−0.126, 0.118	0.949
DHA	0.04	−0.02, 0.10	0.203	0.01	−0.059, 0.072	0.841
EPA+DHA	0.02	−0.01, 0.06	0.250	0.01	−0.040, 0.045	0.914

Bold denotes statistical significance (*p* < 0.05). ALA = α-linolenic acid; BMI = body mass index; CI: confidence interval; DHA = docosahexaenoic acid; EPA = eicosapentaenoic acid; MUFAs = monounsaturated fatty acids; SFAs = saturated fatty acids.

**Table 3:** Unadjusted and adjusted associations between PUFAs and cardiometabolic risk factors.

Variables	Unadjusted OR	95% CI	*p*-Value	Adjusted OR	95% CI	*p*-Value
*Hypertension*						
Omega-6	**0.995**	**0.990, 1.000**	**0.037**	0.998	0.992, 1.004	0.568
ALA	0.978	**0.958, 1.000**	**0.046**	1.008	0.983, 1.033	0.534
EPA	1.005	0.997, 1.014	0.210	1.002	0.993, 1.011	0.640
DHA	**1.004**	**0.999, 1.008**	**0.108**	1.001	0.997, 1.006	0.549
EPA+DHA	**1.002**	**0.999, 1.005**	**0.137**	1.001	0.998, 1.004	0.579
*Diabetes*						
Omega-6	**0.990**	**0.985, 0.995**	**0.001**	**0.993**	**0.986, 0.999**	**0.028**
ALA	**0.941**	**0.919, 0.964**	**0.001**	**0.963**	**0.937, 0.990**	**0.008**
EPA	1.006	0.996, 1.016	0.227	1.004	0.994, 1.014	0.422
DHA	1.004	0.999, 1.009	0.122	1.003	0.997, 1.008	0.344
EPA+DHA	1.003	0.999, 1.006	0.152	1.002	0.998, 1.005	0.368
*Obesity*						
Omega-6	**1.013**	**1.008, 1.018**	**0.001**	0.837	0.038, 18.308	0.910
ALA	**1.039**	**1.015, 1.064**	**0.001**	1.305	0.015, 43.652	0.973
EPA	0.996	0.986, 1.006	0.407	0.967	0.012, 78.358	0.988
DHA	0.996	0.991, 1.001	0.149	0.989	0.065, 14.941	0.989
EPA+DHA	0.998	0.995, 1.001	0.218	0.991	0.185, 5.320	0.992

Bold denotes statistical significance (*p* < 0.05). ALA = α-linolenic acid; BMI = body mass index; CI: confidence interval; DHA = docosahexaenoic acid; EPA = eicosapentaenoic acid; MUFAs = monounsaturated fatty acids; OR = odds ratio; SFAs = saturated fatty acids.

## Data Availability

Raw data files are available from Prof. Landi upon reasonable request. The data are not publicly available due to privacy/ethical restrictions.
